# Supplementary material for: Topology meets superconductivity in a one-dimensional t-J model of magnetic atoms
Source: Nat Commun. 2026 Apr 17;17:5328. doi: 10.1038/s41467-026-71248-8 (PMC13273159; doi:10.1038/s41467-026-71248-8)
Supplement: Supplementary file 1 — Transparent Peer Review file [file 41467_2026_71248_MOESM1_ESM.pdf]

Peer Review File

Topology meets superconductivity in a one-dimensional t-J model of magnetic atoms

Corresponding Author: Mr Leonardo Bellinato Giacomelli

This file contains all reviewer reports in order by version, followed by all author rebuttals in order by version.

Version 0:

Reviewer comments:

Reviewer #1

(Remarks to the Author)
The authors analyze a 1D strongly fermionic interacting model with applications in ultra-cold atoms and lanthanides systems (erbium, dysprosium). Through analytical and numerical DMRG tools, the authors evidence different phases related to superconductivity and topology through the parity and string order parameters in Eqs. (25) and (26). The authors report topological superconducting phases e.g. the TTS phase and establish clear phase diagrams. I find the analysis precise and interesting and I have a few questions to the authors:
- Superconductivity in 1D related to the Luther-Emery Hamiltonian usually occurs as a result of attractive interactions Kc>1 implying also in the spin sector Ks<1 (see e.g. T. Giamarchi's book), as shown through Eqs. (23,24) in the manuscript. Can the authors develop the analysis as a function of typical length of the system related to phase diagram 2 and solutions of RG equations?
- When introducing the string and parity order parameters to classify different phases of matter, how do they relate to usual observables e.g. the same sine and cosine functions usually enter in the singlet-pairing channel, triplet pairing channel, charge density wave orders? This question is e.g. related to the DMRG analysis and SDW and TS channels in Fig. 4. On the vertical axis, it is also not clear what the scale is.
- Related to topological superconductivity, even the LEL phase i.e. Luther-Emery liquid admits zero-energy solutions at the edge in 1D, for open boundaries: see e.g. M. Fabrizio and A. O. Gogolin, arXiv:cond-mat/9504011. Can the authors develop on the edge state analysis as a function of the pinning of the spin bosonic field?
- Related to this, in the discussion section, the authors mention: 'Furthermore, here the edge states are massless in contrast to the massive Majorana fermions occurring in the Kitaev chain', I am not sure I understand this sentence.
- In the same discussion section, the authors mention: `the presence of competing terms U and J_perp is important to see the topological triplet superconductor'. Can the authors be more precise on this interesting observation.
- The authors show a Table with different atoms. What is the typical temperature at which the TTS phase can be seen?

Reviewer #2

(Remarks to the Author)
In the manuscript, the authors describe an approach to simulate a class of 1D extended Hubbard models using magnetic lathanide atoms in optical lattices. The noteworthy result is that in a realistic version, a variety of interesting phases, including a triplet superconducting one with symmetry protected topological order, seems possible. Some of these have observables that are accessible in experiments. The work is of significance to the field of ultracold atoms and quantum simulation, and therefore can belong in nature communications. There are, however, a number of deficiencies that need to be addressed before I can recommend publication. Below, I provide a list of main points to address and minor issues.

Main points:
1) Multi-spin Hubbard models with on-site interactions have for example been studied both theoretically and experimentally with alkaline earth atoms, e.g. in studies with SU(N) interactions. Spin-1/2 bosonic extended Hubbard models with exactly the same form (t-J-V-W models) as the authors proposed have been theoretically proposed before, e.g. in ref[25] where even the interaction range is the same. The authors' model, when restricted to S=1/2, is simply the fermionic verison of the t-J-V-W model with W=0. This should be mentioned, e.g. around eq. 2.

2) The section on "topology and superconducitivty in the low-energy regime" is difficult to read and certain terms are not

defined. Some minor revising of the text is needed.

3) To support the claim of triple superconductivity, Fig 4d needs to somehow include the other correlations functions C_ESS, C_CDW, C_CDW. The Methods only contains this information for specific points. If these other correlators are well below the range shown in the graph, this information can also be captured either in an inset or in the caption.

4) The section on "state preparation and detection scheme" needs expansion both on preparation of low-temperature chains, and also on how observables are detected. Specifically, the authors should address the following issues: a) the proposed method of preparing nbar=1/2 by removing every other particles in a chain will lead to a massive increase in temperature. c) how to remove interchain magnetic coupling is not addressed c) There needs to be a discussion of which observables/diagnostics mentioned in the theory are accessible and how they will be measured. Although string and parity operators seem straightforward in a microscope setup proposed by the authors, this needs to be explicitly spelled out. The authors should mention how they intend to measure pair/pairing correlators (ESS, TS, LSS), which with the exception of LSS are not diagonal in on-site density.

Minor points/question:
1) "this configuration becomes accessible exclusively in ultracold systems of magnetic lanthanide atoms..." -> remove exclusively - seems to strong. Might be true now but can become false in, e.g. if large-scale universal quantum computing becomes possible.
2) "This method, which results effective only in the low energy limit U, Jperp<< T" is a problematic sentence. I think the authors mean "this method is valid in the low energy limit, and when U, Jperp<<T"? Or is the latter condition U,Jperp<<T not required for bosonization?
3) Suggestion for readability: in the sentence "onto a sine gordon model H_SG - Hc+Hs, where", add phrase "onto a Sine-Gordon model H_SG = H_c+H_s, with decoupled charge and spin sectors, where"
4) "this RG analysis allows to determine the relevance of the cosine term in Eq. (3), which occurs when the corresponding field phi_{cs} pins to a constant value". Problematic sentence. I think the authors mean: "this RG analysis allows to determine the relevance of the cosine term in Eq. (3). This term becomes relevant when the corresponding field phi_{cs} is pinned to a constant value, and a finite gap in the cmsm excitation spectrum develops."
5) "phase of matter with such a feature are characterized by the formation of local or nonlocal pairing of fermions with opposite spin." This needs a reference (or a definition of what local/nonlocal pairing means). Otherwise, it is not clear how this follows.
6) "<Szj, ...>" is not clear. The notation <A,B> needs to be defined somewhere.
7) "in addition to the behavior of the correlation functions in Eq. (6) and (8)" - probably good place to start new paragraph.
8) "in contrast, when U>0, and Jperp>0, the bosonization analysis clearly reveals that ...." - remove "clearly".
9) This ties into point (2). Is the bosonization approach only valid when U<<t and Jperp<<t? If so, it would only be valid near the origin of Fig 2a, which states it is a Luttiger Liquid regardless of U/Jperp. This would be inconsistent with the bosonization analysis results.
10) Fig 2(a) missing label "a"
11) "Notably, by further decreasing ... Fig 3(c) indicates that the charge ordering is suddenly replaced". Remove "suddenly" - it does not appear sudden at least on the log-scale plot shown.
12) "a well-known distinctive feature of topological phases is the appearance of edge states, which in a spin-gapped system manifest as finite edge magnetization" - add reference.
13) Fig 4e, add C_ESS, C_SDW ,C_CDW
14) "This unambiguously confirms the presence of a strongly interacting topological phase." Why is it "strongly interacting" and is it relevant that it is?
15) "special attention must also be given to the regime for large repulsive values of Jperp/t" -> replace repulsive with "positive"
16) Fig 5e. What does ESS look like? if it is much smaller, I suggest to either indicate in caption or inset. If it is not much smaller, I suggest to plot it.
17) General question, why is nbar=0.5 picked? How much of this physics survives at other densities?

Reviewer #3

(Remarks to the Author)
Inspired by experimental realization of quantum gases of magnetic lanthanide atoms, the authors investigated novel phases of such systems in 1D optical lattice. The strong magnetic dipolar interaction results in a modified t-J model. The original t-J model is obtained as an effective description of the Hubbard model in the strong interaction limit, in which an effective Heisenberg coupling with strength ~ t^2/U (t and U being the single particle hopping and on-site interaction, respectively) can be obtained.

A key advantage of the current set up is that the magnetic coupling strength originates from the magnetic dipolar interaction and, hence, can be independently tuned with respect to t and U. This flexibility allows one to reach parameter regimes not possible with Hubbard model.

Through bosonization and the DMRG calculation, it is shown that the modified t-J model does feature a very rich phase diagram, including different topological and superconducting phases.

I have a few comments I think the authors should address:
(1) The results presented are obtained at zero temperature. It is mentioned in the end that these phases can be realized for

temperatures below the spin gap, i.e., T < 0.2 t. This cannot be true for all the phases, as some of which have vanishing spin gap. Even for certain phases with non-zero spin gap (e.g., TTS, TL), this may not be true. A more careful discussion of finite temperature effects is needed.
(2) It is discussed that quantum gas microscopy will be employed to detect various phases. Is this sufficient to detect the triplet superconducting phases such as LTS and TTS?
(3) The calculation is done under the zero total magnetization. Can the authors comment on finite magnetization. In particular, which of the phases are sensitive to small spin imbalance?

Some minor comments:
(1) Is the average particle number (\bar{n}) equal to 1/2 for Fig.2, like the rest of the figures? It should be indicated in the figure caption.
(2) The notation in Eqs.(8) and (9), <... , ...> should be explained.

Version 1:

Reviewer comments:

Reviewer #1

(Remarks to the Author)
I have read the new version of the article and the responses to the questions of the three Referees. The authors have done a nice work with the changes and they respond satisfactorily to all the questions. Therefore, I recommend publication of the article in Nature Communications.

But, I have one last request addressed to the authors: related to my question 3 as Referee 1, can the authors develop the physical meaning of Sz^edge=1/4 in terms of quasiparticles understanding. The authors provide a justification in terms of pinning of a bosonic field at a certain value. The authors (only) mention briefly: “This implies that the SPT phases are characterized by the LRO of the string correlation function as well as the appearance of protected edge modes.” but they do not elaborate on the precise meaning of this result nor on physical consequences.

Reviewer #2

(Remarks to the Author)
The revised manuscript has addressed my previous comments adequately. In particular, the authors have substantially bolstered the section "state preparation and detection scheme.” While the issue of whether low enough entropies can be reached is not fully addressed, I agree with the authors that this should be left to future work. Optimal schemes to prepare low entropy Fermi gases in lattice is an open experimental question under active research.

I can recommend publication with the minor corrections/modifications below:

1)",with decoupled charge and spin sectors", missing space after comma
2)"Guaranty" -> modify relevant sentence to be along the lines of “to suppress interactions between different rows” (whether it is neighboring interactions at 1/8 strength is "negligible" should be left to future studies)

Reviewer #3

(Remarks to the Author)
I find the authors' responses to all reviewers' comments satisfactory. I can now recommend its publication.

Open Access This Peer Review File is licensed under a Creative Commons Attribution 4.0 International License, which permits use, sharing, adaptation, distribution and reproduction in any medium or format, as long as you give appropriate credit to the original author(s) and the source, provide a link to the Creative Commons license, and indicate if changes were made.
In cases where reviewers are anonymous, credit should be given to 'Anonymous Referee' and the source.
The images or other third party material in this Peer Review File are included in the article's Creative Commons license, unless indicated otherwise in a credit line to the material. If material is not included in the article's Creative Commons license and your intended use is not permitted by statutory regulation or exceeds the permitted use, you will need to obtain permission directly from the copyright holder.
To view a copy of this license, visit https://creativecommons.org/licenses/by/4.0/

Response to Referees: NCOMMS-25-66547

L. Bellinato Giacomelli, T. Bland, L. Lafforgue, F. Ferlaino, M. J. Mark and L. Barbiero

January 1, 2026

We thank the Editor and the Referees for the time and effort devoted to evaluating our manuscript. We believe that the suggested changes have significantly improved the quality and clarity of the paper. In the revised manuscript, as well as in the present response, we address all comments and questions raised by the three referees. We think that, in light of these revisions, the manuscript is now suitable for publication in Nature Communications.

In this response as well as in the revised manuscript we have highlighted in blue the answers to the comments/questions as well as the changes made to the manuscript.

1 Referee #1

We thank the referee for the careful reading and the positive assessment of our manuscript. In the following, we address all the points raised by the referee.

The authors analyze a 1D strongly fermionic interacting model with applications in ultra-cold atoms and lanthanides systems (erbium, dysprosium). Through analytical and numerical DMRG tools, the authors evidence different phases related to superconductivity and topology through the parity and string order parameters in Eqs. (25) and (26). The authors report topological superconducting phases e.g. the TTS phase and establish clear phase diagrams. I find the analysis precise and interesting and I have a few questions to the authors:

1. *Superconductivity in 1D related to the Luther-Emery Hamiltonian usually occurs as a result of attractive interactions $K_c > 1$ implying also in the spin sector $K_s < 1$ (see e.g. T. Giamarchi's book), as shown through Eqs. (23,24) in the manuscript. Can the authors develop the analysis as a function of typical length of the system related to phase diagram 2 and solutions of RG equations?*

The bosonization analysis presented in this manuscript is performed under the assumption of an infinite chain $L \to \infty$, i.e. in the thermodynamic limit (TDL). For the numerical simulations, we use a chain length of $L = 240$, as it provides an excellent approximation of the thermodynamic limit. In order to prove this point, we have added as an example Fig.10, where we present the finite-size scaling analysis of the Luttinger parameter K_c shown in the inset of Fig.3c of the main text manuscript. This new Figure shows that a thermodynamic extrapolation gives rise to corrections of the order of 10^{-2} with respect to the values obtained at the finite size $L = 240$. For sizes sensibly smaller than our chosen one, the transition points become not properly defined and the extension of the different phases strongly depends on L. Although we believe that this point is of certain interest especially for experimental purposes, we think that this is not so crucial to the scope of the present manuscript. Finally, we note that close to the origin of our phase diagram (Fig.2a), the bosonization analysis captures all the phases that we find through the DMRG analysis.

2. *When introducing the string and parity order parameters to classify different phases of matter, how do they relate to usual observables e.g. the same sine and cosine functions usually enter in the singlet-pairing channel, triplet pairing channel, charge density wave orders? This question is e.g. related to the DMRG analysis and SDW and TS channels in Fig. 4. On the vertical axis, it is also not clear what the scale is.*

   Following this interesting point, we expanded the subsection "Bosonization analysis". This now includes the bosonized expression of all the order parameters entering in the definitions of the correlation functions that we use to characterize the leading orders. In this way, the relation between the correlations and the bosonic fields $\phi_{c,s}$ is explicit.

   Regarding Fig. 4 subplot (e), as pointed out in the caption, we plot the asymptotic value of the correlators C_{SDW} and C_{TS} calculated over a distance of $r = 162$ sites in a chain of $L = 240$ so to minimize boundary effects which can alter the correlation function decay. In addition, in the new version of this figure, we also added the asymptotic values of the charge density wave C_{CDW} and extended singlet superconducting C_{ESS} correlations so to explicitly show the leading order.

3. *Related to topological superconductivity, even the LEL phase i.e. Luther-Emery liquid admits zero-energy solutions at the edge in 1D, for open boundaries: see e.g. M. Fabrizio and A. O. Gogolin, arXiv:cond-mat/9504011. Can the authors develop on the edge state analysis as a function of the pinning of the spin bosonic field?*

   We thank the referee for raising this important point. We first point out that, as discussed in arXiv:cond-mat/9504011, in the LEL, the appearance of zero-energy solutions reflects the presence of edge bound states formed by fermionic pairs. In our case, the appearance of edge

solutions, which are not bound states, are related to the presence of the SPT phases. Following the analysis in [55], we find that these edge solutions appear when ϕ_s is pinned to $\pm\sqrt{\pi/8}$ and ϕ_c is unpinned. This can be seen by integrating the smooth component of the onsite spin density, given by $S^z_j \sim \frac{d_x}{2\pi}\partial_x\phi_s(x)$, at the edge of the system. The calculation gives $S^z_{edge} = \lim_{d_x\to 0}\frac{1}{\sqrt{2\pi}}\int_{x_e}^{x_e+d_x} dx\partial_x\phi_s(x) = \pm\frac{1}{4}$, where we take the limit $d_x \to 0$ to find the accumulated spin at the edge x_e. We have highlighted this point in the section "Topology and superconductivity in the low-energy regime" of the manuscript.

4. *Related to this, in the discussion section, the authors mention: 'Furthermore, here the edge states are massless in contrast to the massive Majorana fermions occurring in the Kitaev chain', I am not sure I understand this sentence.*

   We thank the referee for asking about this fundamental point which we realized was not properly explained in the text. What we wanted to point out here was that we do not find any signature of edge states/bulk Majorana fermions in our TSS phase. In the new version of the text we changed the sentence accordingly.

5. *In the same discussion section, the authors mention: 'the presence of competing terms U and $J_\perp$ is important to see the topological triplet superconductor'. Can the authors be more precise on this interesting observation.*

   We first underline that the SPT order that we unveil in our manuscript does correspond to a spin gapped regime characterized by hidden antiferromagnetic order where up and down fermions are always alternated and separated by an arbitrary number of empty or doubly occupied sites. In this regard, it is important to analize separately the roles played by the terms in our Hamiltonian: the tunneling t favors the particle delocalization; the spin-flip term $J_\perp$ promotes configurations where up and down spins are localized in consecutive sites forming non-local pairs; positive/negative values of U make energetically favorable states with high number of singly and empty/doubly occupied sites. That said, for positive and intermediate values of $J_\perp$ the competition between the tunneling t and the spin-flip allows the fermions to be diluted and alternated and therefore to form an effective hidden antiferromagnet, i.e. the SPT order. When adding to this picture an attractive U, we find that the wave function overlap is favored and therefore superconductivity can emerge while keeping untouched the hidden antiferromagnetic SPT order. From a bosonization point of view, we see that the bosonic field ϕ_s remains pinned to $\pm\sqrt{\pi/8}$, which is precisely the value for which the string correlation function acquires LRO, edge states are formed and a spin gap is open, for both attractive and repulsive U. However, the condition implying superconductivity $K_c > 1$ occurs only when U becomes negative. As we now explicitly discuss in the subsection "Bosonization analysis", a closer investigation shows

that the dominant instability is the triplet superconducting one, thus confirming the numerical results. Furthermore, with this statement we also wish to highlight the fact that differently from the Kitaev-chain, in which superconductivity is explicitly induced through a term of the form $(\Delta^* c_i^\dagger c_{i+1}^\dagger + H.c.)$, in our model it arises from the competition between the onsite interaction U and the spin-flip interaction $J_\perp$.

6. *The authors show a Table with different atoms. What is the typical temperature at which the TTS phase can be seen?*

   We thank the referee for this important question. The temperatures needed to observe the topological triplet superconductor are determined by the ratio between the spin gap Δ_s and the hopping t. More specifically, the system has to be "colder than the gap". This means that the temperature T in units of hopping t has to be smaller than Δ_s/t. Depending on the specific hopping amplitude and gap, this then translates into a specific temperature. The coldest temperature that has been reached in an experiment is $T/t \sim 0.05$ [81], which is a temperature far below the gap we find in the local as well as extended singlet superconducting phases, and approximately four times smaller than the gap we find in the topological superconductor. Importantly, the gaps we find grow as the system size reduces, thus making experimental realizations easier.

2 Referee #2

We thank the referee for the careful reading and the positive assessment of our manuscript. In the following, we address all the points raised by the referee.

In the manuscript, the authors describe an approach to simulate a class of 1D extended Hubbard models using magnetic lathanide atoms in optical lattices. The noteworthy result is that in a realistic version, a variety of interesting phases, including a triplet superconducting one with symmetry protected topological order, seems possible. Some of these have observables that are accessible in experiments. The work is of significance to the field of ultracold atoms and quantum simulation, and therefore can belong in nature communications. There are, however, a number of deficiencies that need to be addressed before I can recommend publication. Below, I provide a list of main points to address and minor issues.
Main points:

1. *Multi-spin Hubbard models with on-site interactions have for example been studied both theoretically and experimentally with alkaline earth atoms, e.g. in studies with SU(N) interactions. Spin-1/2 bosonic extended Hubbard models with exactly the same form (t-J-V-W models) as the authors proposed have been theoretically proposed before, e.g. in ref[25] where even the interaction range is the same. The authors' model, when restricted to S=1/2, is simply the fermionic*

verison of the t-J-V-W model with W=0. This should be mentioned, e.g. around eq. 2.

We thank the referee for this comment. We have amended the main text and better highlighted how our model relates to the t–J model derived in [25] as well as in studies with SU(N) interactions, see the added reference [33].

2. *The section on "topology and superconductivity in the low-energy regime" is difficult to read and certain terms are not defined. Some minor revising of the text is needed.*

   We have ensured that all quantities are defined, and have extended the analysis to include a discussion on the appearance of edge modes in the SPT regime. We believe that this, combined with the suggestions made by the referees has improved the section "Topology and superconductivity in the low-energy regime".

3. *To support the claim of triple superconductivity, Fig 4d needs to somehow include the other correlations functions C_{ESS}, C_{CDW}, C_{CDW}. The Methods only contains this information for specific points. If these other correlators are well below the range shown in the graph, this information can also be captured either in an inset or in the caption.*

   We have added the charge density wave (CDW) and extended singlet superconducting (ESS) correlation functions to Fig.4e.

4. *The section on "state preparation and detection scheme" needs expansion both on preparation of low-temperature chains, and also on how observables are detected. Specifically, the authors should address the following issues: a) the proposed method of preparing $\bar{n} = 1/2$ by removing every other particles in a chain will lead to a massive increase in temperature. b) how to remove interchain magnetic coupling is not addressed c) There needs to be a discussion of which observables/diagnostics mentioned in the theory are accessible and how they will be measured. Although string and parity operators seem straightforward in a microscope setup proposed by the authors, this needs to be explicitly spelled out. The authors should mention how they intend to measure pair/pairing correlators (ESS, TS, LSS), which with the exception of LSS are not diagonal in on-site density.*

   We thank the referee for these fundamental questions.

   (a) *We believe that our proposed preparation method - initializing a Band insulator of spin-polarized fermions, removing atoms from every 2nd lattice site to create a charge-density wave, and subsequent antiferromagnetic spin preparation - will bring us close to the ground state for a frozen system, before entering the phase diagram by adiabatically changing the system parameters. Nonetheless, further investigations might be needed to theoretically*

find the best strategy to reach the different phases found in this work, which we believe goes beyond the current scope of the paper.

(b) *Interchain coupling can be greatly reduced by increasing the distance between them, either via emptying chains via local addressing or increasing the lattice site distance itself i.e. $d_y \gg d_x$ (already $d_y = 2d_x$ will reduce interchain coupling by about one order of magnitude). We now added a short discussion to the text.*

(c) *The proposed quantum gas microscope setup should allow local spin-resolved density measurements. This will allow us to measure charge and spin density correlations as well as edge state magnetization. To measure pairing correlations we envision the usage of a superlattice together with global rotations similar to recent work performed already in experiments [75]. More recent work [76] has further investigated such protocols and developed a new toolbox. We have added this information to the manuscript.*

Minor points/question:

1. *"this configuration becomes accessible exclusively in ultracold systems of magnetic lanthanide atoms..." → remove exclusively - seems to strong. Might be true now but can become false in, e.g. if large-scale universal quantum computing becomes possible.*

   We have removed the word exclusively.

2. *"This method, which results effective only in the low energy limit U, $J_\perp << T$" is a problematic sentence. I think the authors mean "this method is valid in the low energy limit, and when U, $J_\perp << T$"? Or is the latter condition U,$J_\perp << T$ not required for bosonization?*

   Indeed, the Bosonization approach relies on a continuum description of the microscopic Hamiltonian, which is based on the idea of linearizing the dispersion relation of the hopping term near the two symmetric Fermi momenta $\pm k_F$. In our treatment we then consider interactions perturbatively, i.e., we work in the weak-coupling regime U, $J_\perp \ll t$. We have modified the main text to make this point clearer.

3. *Suggestion for readability: in the sentence "onto a sine gordon model $H_{SG} = H_c + H_s$, where", add phrase "onto a Sine-Gordon model $H_{SG} = H_c + H_s$, with decoupled charge and spin sectors, where"*

   We thank the referee for the suggestion. We have changed the text accordingly.

4. *"this RG analysis allows to determine the relevance of the cosine term in Eq. (3), which occurs when the corresponding field $\phi_{c,s}$ pins to a constant value". Problematic sentence. I think the authors mean: "this RG analysis allows to determine the relevance of the cosine term in Eq.*

(3). This term becomes relevant when the corresponding field $\phi_{c,s}$ is pinned to a constant value, and a finite gap in the cmsm excitation spectrum develops."

We thank the referee for the suggestion. We have changed the text accordingly.

5. *"phase of matter with such a feature are characterized by the formation of local or nonlocal pairing of fermions with opposite spin." This needs a reference (or a definition of what local/nonlocal pairing means). Otherwise, it is not clear how this follows.*

   Local pairing refers to the formation of pairs with opposite spins ($m_F = \pm 1/2$) on a given site, whereas nonlocal pairs, while still having opposite spins, are formed by particles which reside on different lattice sites. We have added two references [46,48] to the main text which clarify this statement.

6. *"$< Szj, ... >$" is not clear. The notation needs to be defined somewhere.*

   We thank the referee for noticing these typos. We have removed the comma from Eqs.(8,9,14,15).

7. *"in addition to the behavior of the correlation functions in Eq. (6) and (8)" - probably good place to start new paragraph.*

   We agree, we now start a new paragraph.

8. *"in contrast, when $U > 0$, and $J_\perp > 0$, the bosonization analysis clearly reveals that ...." - remove "clearly".*

   We have removed the word clearly.

9. *This ties into point (2). Is the bosonization approach only valid when $U << t$ and $J_\perp << t$? If so, it would only be valid near the origin of Fig 2a, which states it is a Luttiger Liquid regardless of $U/J_\perp$. This would be inconsistent with the bosonization analysis results.*

   It is indeed expected that in the weak-coupling regime $U, J_\perp \ll t$ the physics of the system can be well understood by firstly going to a continuum description of the problem and then performing a Bosonization analysis. Based on these approximations, this suggests that the results obtained through Bosonization should identify the phases and the transition points of our model close to the origin of Fig.2a. Although not subject to these approximations, DMRG not always allows for an accurate determination of the transition points. This aspect is particularly true for phase transitions where one gap opens exponentially and therefore the precise identification of the boundary between two phases becomes highly challenging. Importantly, this is exactly

the scenario that we face when we study the LL-TL phase transition. Therefore, rather that considering the point raised by the referee as an inconsistency, we believe that this behavior is just a signature of the DMRG and Bosonization limitations when studying such specific phase transitions.

10. *Fig 2(a) missing label "a"*

    We have added the label (a) when specifically referring to the phase diagram of Fig.2 in the main text, as well as improving the visibility of the label "a" in Fig.2 itself.

11. *"Notably, by further decreasing ... Fig 3(c) indicates that the charge ordering is suddenly replaced". Remove "suddenly" - it does not appear sudden at least on the log-scale plot shown.*

    We have removed the word "suddenly".

12. *"a well-known distinctive feature of topological phases is the appearance of edge states, which in a spin-gapped system manifest as finite edge magnetization" - add reference.*

    We have added references [49] and [55] to support this statement.

13. *Fig 4e, add C_{ESS}, C_{SDW} ,C_{CDW}*

    We have added the charge density wave (CDW) and extended singlet superconducting (ESS) correlation functions to Fig.4e while C_{SDW} was already there.

14. *"This unambiguously confirms the presence of a strongly interacting topological phase." Why is it "strongly interacting" and is it relevant that it is?*

    We understand that "strongly interacting" in this context can be misleading. We have changed the text to "interaction induced" as this better captures the intent of our statement.

15. *"special attention must also be given to the regime for large repulsive values of $J_\perp/t$" $\rightarrow$ replace repulsive with "positive"*

    We have replaced "repulsive" with "positive".

16. *Fig 5e. What does ESS look like? if it is much smaller, I suggest to either indicate in caption or inset. If it is not much smaller, I suggest to plot it.*

    We have added the extended singlet superconducting (ESS) correlation function to Fig.5e.

17. *General question, why is $\bar{n} = 0.5$ picked? How much of this physics survives at other densities?*

Indeed the bosonization analysis in the text as well as the phase diagram presented in Fig.2 are for a density of $\bar{n} = 0.5$. There is no specific reason for this choice. In order to study the stability of these phases with respect to density variations we present in the main text a second phase diagram (Fig.6) as a function of the onsite interaction U/t and the density $\bar{n}$, for fixed $J_\perp/t = 1.25$. The choice of this specific $J_\perp/t$ is to remain consistent with the panels shown in Fig.5. From this analysis, we observe that both the topological phase and the topological superconducting phase exhibit exceptional stability against density variations. Moreover, the bosonization analysis supports these findings, as it reveals that the field ϕ_s remains pinned to $\pm\sqrt{\pi/8}$ for $U > -2.5$ and to 0 for $U < -2.5$, across the entire range of densities $0 < \bar{n} < 1$. Furthermore, the Luttinger parameter K_c is larger than unity when $\phi_s = 0$, as well as a region in which the pinning changes to $\phi_s = \pm\sqrt{\pi/8}$. Thus, our bosonization analysis predicts the emergence of both the topological liquid phase and topological superconducting phase across the entire range of densities, consistent with the results we obtain from the DMRG analysis. Finally, we observe that as for the numerical results, the regime in which we find the topological triplet superconductor, expands with increasing $\bar{n}$.

3 Referee #3

We thank the referee for the careful reading and the positive assessment of our manuscript. In the following, we address all the points raised by the referee.

Inspired by experimental realization of quantum gases of magnetic lanthanide atoms, the authors investigated novel phases of such systems in 1D optical lattice. The strong magnetic dipolar interaction results in a modified t-J model. The original t-J model is obtained as an effective description of the Hubbard model in the strong interaction limit, in which an effective Heisenberg coupling with strength $\sim t^2/U$ (t and U being the single particle hopping and on-site interaction, respectively) can be obtained.

A key advantage of the current set up is that the magnetic coupling strength originates from the magnetic dipolar interaction and, hence, can be independently tuned with respect to t and U. This flexibility allows one to reach parameter regimes not possible with Hubbard model.

Through bosonization and the DMRG calculation, it is shown that the modified t-J model does feature a very rich phase diagram, including different topological and superconducting phases.

I have a few comments I think the authors should address:

1. *The results presented are obtained at zero temperature. It is mentioned in the end that these*

phases can be realized for temperatures below the spin gap, i.e., $T < 0.2t$. This cannot be true for all the phases, as some of which have vanishing spin gap. Even for certain phases with non-zero spin gap (e.g., TTS, TL), this may not be true. A more careful discussion of finite temperature effects is needed.

We thank the referee for raising this interesting point. In the discussion section at the end of the main text we highlight the very important fact that our setup only requires temperatures which lie below the spin gap Δ_s. Specific to the topological phase as well as the topological superconducting phase, this corresponds to $T/t \lesssim 0.2$ (value which we specify in the main text), whereas for the other superconducting phases the spin gap is larger, thus allowing for larger T/t. We further underline that experiments operate at finite size where in general gaps are always finite although potentially very small even in thermodynamically gapless phases. We thus expect our phase diagram to remain rather stable with respect to typical thermal fluctuations occurring in ultracold atoms experiments.

2. *It is discussed that quantum gas microscopy will be employed to detect various phases. Is this sufficient to detect the triplet superconducting phases such as LTS and TTS?*

   The proposed quantum gas microscope setup should allow local spin-resolved density measurements. This will allow us to measure charge and spin density correlations as well as edge state magnetization. To measure pairing correlations we envision the usage of a superlattice together with global rotations similar to recent work performed already in experiments [75]. More recent work [76] has further investigated such protocols and developed a new toolbox. We have added this information to the manuscript.

3. *The calculation is done under the zero total magnetization. Can the authors comment on finite magnetization. In particular, which of the phases are sensitive to small spin imbalance?*

   We thank the referee for raising this point. As shown in a similar model [65], in which analogous phases arise due to different processes, we expect that the spin imbalance gives rise to phase separation. In such a scenario, the system results characterized by a region where the regimes occurring at vanishing total magnetization still take place, and regions where the exceeding fermions form a fully polarized Luttinger liquid. We further stress that if only one spin is added, the system remains in the topological phase as, in analogy for instance to the Haldane phase in XXZ S=1 phase or the SSH model, the topological phase is stable also in the sector of total magnetization ± 1.

Some minor comments:

1. *Is the average particle number ($\bar{n}$) equal to 1/2 for Fig.2, like the rest of the figures? It should be indicated in the figure caption.*

We thank the referee for this suggestion. Indeed the phase diagram of Fig.2 is for density $\bar{n} = 0.5$. We have added this information to the caption.

2. *The notation in Eqs.(8) and (9), $< ..., ... >$ should be explained.*

   We thank the referee for bringing this typo to our attention. We have corrected the typo.

We wish to thank the referees once more for the very interesting comments and suggestion. We are confident that the changes made have greatly strengthened the manuscript.

4 List of changes

1. *We have added to the methods the section* ***Extrapolation of K_c to the thermodynamic limit*** *which includes a new figure (Fig.10).*

2. *As suggested by the referees we have added to Fig.4e the charge density wave (CDW) correlation function and to Figs.4e and 5e the extended singlet superconducting (ESS) correlator.*

3. *We have enlarged the subplot labels (a-e) in Fig.2 and changed the color of label (a) for better visibility.*

4. *We expanded the subsection* ***Bosonization analysis*** *in the Methods to include the expressions of all the order parameters entering in the definitions of the correlation functions that we use to characterize the leading orders.*

5. *In the subsection* ***Topology and superconductivity in the low-energy regime*** *we now added a discussion on the edge states.*

6. *Change of text: changed "Furthermore, here the edge states are massless in contrast to the massive Majorana fermions occurring in the Kitaev chain." to "Furthermore, here we do not have any evidence that the edge states are Majorana fermions".*

7. *Added: It is fair to mention that for $U \gg t$, i.e. where the state $|-1/2, 1/2\rangle$ is prohibited, Eq.2 shares similarities with polar molecules setups [25].*

8. *Added: We underline that higher-spin t–J models with preserved rotational symmetry can also be realized in SU(N) systems [33].*

9. *Added: In practice we intend to have $d_y > 2d_x$ to guaranty that the magnetic interactions between the different rows is negligible.*

10. *Added: In addition, the use of a superlattice in combination with global addressing techniques allows for the measurement of pairing correlators [75,76]*

11. *Added two references [46,48] to clarify meaning of local and nonlocal pairing.*

12. *Added references [49] and [55] to support statement: "a well-known distinctive feature of topological phases is the appearance of edge states, which in a spin-gapped system manifest as finite edge magnetization".*

13. *Added extended singlet superconducting correlation function to Fig.5e.*

Additional minor changes are highlighted in blue in the revised manuscript as well as reported in the point-by-point reply above. We have also corrected minor typos which we have spotted during the review process.

2nd Response to Referees: NCOMMS-25-66547

L. Bellinato Giacomelli, T. Bland, L. Lafforgue, F. Ferlaino, M. J. Mark and L. Barbiero

March 3, 2026

We sincerely thank the Editor and the Referees for their positive review of our revised manuscript and, in particular, for their insightful questions and comments, which have significantly improved the quality of our work. In response to Referee 1, we have added a brief discussion to the main text regarding the fractional edge magnetization and its direct quasiparticle interpretation. Furthermore, in the new version of the manuscript we also address the comments of referee 2.

In the revised manuscript we have highlighted in orange the changes made.
